# Congruent Genetic and Demographic Dispersal Rates in a Natural Metapopulation at Equilibrium

**DOI:** 10.3390/genes12030362

**Published:** 2021-03-03

**Authors:** Delphine Legrand, Michel Baguette, Jérôme G. Prunier, Quentin Dubois, Camille Turlure, Nicolas Schtickzelle

**Affiliations:** 1 Theoretical and Experimental Ecology Station (UMR 5371), National Centre for Scientific Research (CNRS), Paul Sabatier University (UPS), 09200 Moulis, France; michel.baguette@mnhn.fr (M.B.); jerome.prunier@gmail.com (J.G.P.); 2 Institut Systématique, Evolution, Biodiversité (ISYEB), UMR 7205 Museum d’Histoire Naturelle, CNRS, Sorbonne Université, EPHE, Université des Antilles, F-75005 Paris, France; 3 Biodiversity Research Centre, Earth and Life Institute, Université Catholique de Louvain, 1348 Louvain-la-Neuve, Belgium; quent.dubois@gmail.com (Q.D.); turlure_camille@hotmail.com (C.T.); nicolas.schtickzelle@uclouvain.be (N.S.)

**Keywords:** butterfly metapopulation, dispersal, genetic structure, demography, spatio-temporal stability, environmental fluctuations, *Boloria eunomia*

## Abstract

Understanding the functioning of natural metapopulations at relevant spatial and temporal scales is necessary to accurately feed both theoretical eco-evolutionary models and conservation plans. One key metric to describe the dynamics of metapopulations is dispersal rate. It can be estimated with either direct field estimates of individual movements or with indirect molecular methods, but the two approaches do not necessarily match. We present a field study in a large natural metapopulation of the butterfly *Boloria eunomia* in Belgium surveyed over three generations using synchronized demographic and genetic datasets with the aim to characterize its genetic structure, its dispersal dynamics, and its demographic stability. By comparing the census and effective population sizes, and the estimates of dispersal rates, we found evidence of stability at several levels: constant inter-generational ranking of population sizes without drastic historical changes, stable genetic structure and geographically-influenced dispersal movements. Interestingly, contemporary dispersal estimates matched between direct field and indirect genetic assessments. We discuss the eco-evolutionary mechanisms that could explain the described stability of the metapopulation, and suggest that destabilizing agents like inter-generational fluctuations in population sizes could be controlled by a long adaptive history of the species to its dynamic local environment. We finally propose methodological avenues to further improve the match between demographic and genetic estimates of dispersal.

## 1. Introduction

The metapopulation concept provides an operational framework for both (evolutionary) ecologists and conservation managers [1]. Classically defined as a set of interacting populations for which frequent local extinctions are balanced by recolonization [2,3], a metapopulation can also broadly refer to patchy populations [4], that is, to any set of local populations potentially related by movements of individuals in a landscape [5]. While retaining the fundamental aspect of the classical metapopulation concept, i.e., a biological structure linking local and regional-scale processes, the latter definition reflects a wider variety of biological situations (e.g., [6,7]). Classical metapopulations indeed correspond to a narrow range of ecological parameters (i.e., patch occupancy, turnover, etc., [8]) and many metapopulations do not experience local population extinctions at each generation [7]. In any case however, describing the long-term functioning of natural metapopulations, a necessary step to accurately predict how they would respond to contemporary environmental changes, relies on an accurate knowledge of the dispersal process. 

Dispersal, individual movements potentially leading to gene flow [9,10], is the eco-evolutionary process creating biological links within metapopulations [11], and thereby within ecological networks [12]. It affects local adaptation (either positively or negatively, [13,14]) and spatial synchrony among populations [15], buffers the risk of local extinctions through (genetic) rescue effects [16] and allows recolonization of vacant patches [4]. As such, dispersal is central for metapopulation stability [17]. Any biotic or abiotic change occurring at the local or regional scale and affecting dispersal (e.g., landscape modification, decrease in individuals’ movement ability) may result in the collapse of the whole metapopulation. Both the frequent short-distance and the rarest long-distance dispersal movements may have a significant impact on metapopulations’ equilibrium [18]. Dispersal rate is thus a fundamental metric of metapopulation functioning, which needs to be measured at accurate spatial and temporal scales in nature [19].

Dispersal rate is the proportion of individuals that move from a population to another. It can be estimated on the field through two approaches relying on distinct theoretical frameworks: demography and genetics. Demographic estimates of dispersal rates are obtained either through the direct monitoring of individual movements using visual observation or telemetric systems depending on landscapes and taxa [20,21,22], or through Capture-Mark-Recapture (CMR) approaches [23]. From such movement data a dispersal kernel can be computed, i.e., the probability density function that dispersing individuals move a certain distance. The quality of dispersal kernels, a key element to describe and predict the dynamics of a metapopulation, is strongly dependent on the quality of the raw movement data, but there are a number of limitations with these direct measurements of dispersal rates. First, they often require laborious and costly field sessions [24,25]. Second, there is a recurrent bias toward the recording of short-distance movements to the detriment of long-distance movements, meaning that dispersal kernels might only model processes occurring at small spatial scales (see [26] for an example of a mathematical correction of biased dispersal kernels). Third, dispersal is context-dependent, which means that “the existence of a species-specific dispersal function is probably a myth” [27]. This poses limits to the transfer of dispersal kernels, even between metapopulations of the same species (e.g., [28]). Finally, whatever the precision of the recording of dispersal movements and hence of dispersal kernels, direct measures of dispersal do not provide information on how much the movements contribute to the gene pool at the next generations. However, reliable estimates of effective dispersal are key to understand the evolutionary dynamics and long-term stability of metapopulations.

The genetic approach indirectly estimates effective dispersal rates through its impact on the genetic structure at the metapopulation level, determined from allelic composition and frequencies in the local populations (from microsatellites, Amplified Fragment Length Polymorphisms AFLPs, Single-Nucleotid Polymorphisms SNPs, etc.). Most often, the genetic differentiation index *F_ST_* is used as a proxy for historical effective number of dispersers (often called “migrants” in the genetic literature) between pairs of populations [29]. A series of unrealistic assumptions, including symmetrical fluxes and equal population sizes, are however required to apply Wright’s island model [30]. Refinements have been proposed (e.g., [31]), and coalescent-based methods have notably been developed to directly infer asymmetrical effective dispersal rates (see reviews in, e.g., [12,32,33]). The choice between these different methods often results from a balance between accepting some violation of model assumptions and analysis complexity (determining the time required to run the analysis). Since genetic estimates of dispersal rates might not reflect contemporary gene flow, especially because of the time lag between the contemporary processes affecting dispersal and the actual setting-up of genetic differentiation among populations [34], methods such as Bayesian assignment (e.g., [35,36]) or parentage analyses [37] have been developed to identify dispersers among individual genotypes. As for direct observation of movements, these latter methods inform on the origin and the target populations of each detected disperser. Nonetheless, as for demography, estimates of effective dispersal present some limitations. For instance, and as discussed above, natural situations rarely fulfill all genetic models’ assumptions. Furthermore, indirect measures of dispersal do not inform on the phenotypic traits of dispersers. It thus has recurrently been proposed to combine, when possible, demographic and genetic approaches to understand metapopulation dynamics and estimate their stability over time (e.g., [12,25,32,33,38]). Such integrative studies remain however rare, and there is a general call for empiricists to compare dispersal estimates with different methodologies at appropriate spatio-temporal scales ([12,32], see [25,39] for examples).

Here, we present a field study in which we analyzed the population structure, the dynamics, and the stability of a natural butterfly metapopulation in the Belgian Ardenne combining demographic and genetic datasets obtained at a large spatial scale (~200 km^2^) over three generations (three years). The bog fritillary *Boloria eunomia* has long been used as a model species in the metapopulation literature because of its patchy distribution and sensitivity to habitat fragmentation. Over the last three decades, important knowledge has accumulated regarding *B. eunomia* habitat use [40], dispersal behaviour and demography [41,42,43,44], metapopulation functioning [45,46,47,48]), and genetic structure at local, regional and continental scales [49,50,51,52]. However, none of these studies have synthesized both demographic and genetic data within the same large network of populations over several generations. Our objectives were thus to:

(i) determine the genetic structure within a *B. eunomia* metapopulation based on genetic material collected on more than 1000 individuals over three generations across nine local populations;
(ii) describe the dynamics of the whole-metapopulation through a thorough comparison between genetic and demographic estimates of dispersal rates;
(iii) evaluate the long-term demographic stability of the metapopulation.

Despite important inter-annual fluctuations in census population sizes, local population extinctions were very rarely observed over two decades of field sampling in this metapopulation [45]. We thus predicted that the metapopulation should harbour high degree of genetic stability, both in terms of genetic structure and long-term effective population sizes. Predictions are more uncertain regarding the congruence between direct and indirect dispersal estimates. We predicted that the correlation between genetic and demographic estimates of dispersal should be strong only provided that: (i) the assumptions of demographic and genetic models are not largely violated within the studied *B. eunomia* metapopulation, (ii) long-distance dispersal movements, particularly difficult to record in the field, are not too frequent; (iii) a relatively high proportion of the dispersal movements are effective in terms of gene flow among populations.

## 2. Materials and Methods

### 2.1. Model Species and Study Area

**Figure 1:**
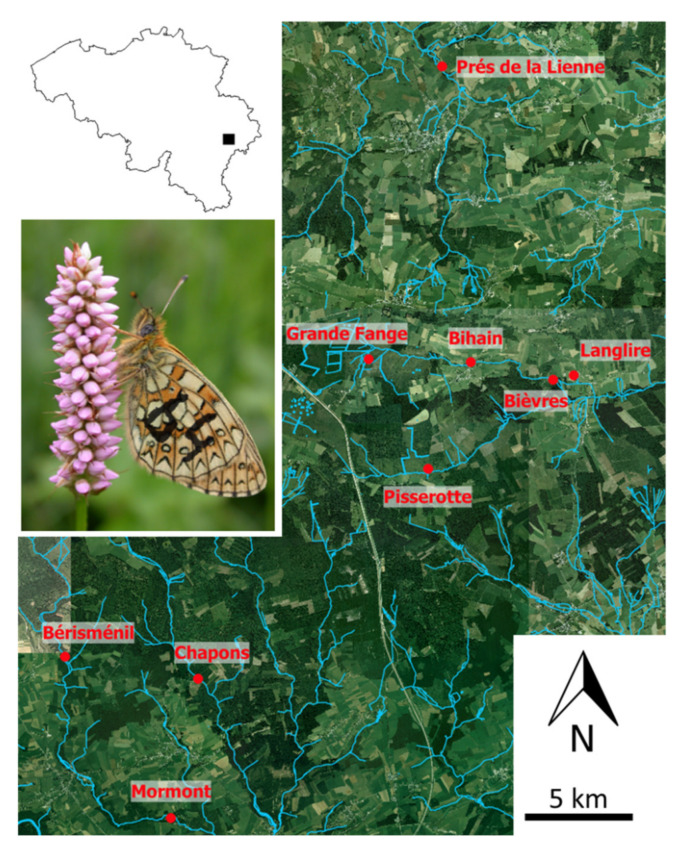
Model species and study area. The studied metapopulation is situated in the Ardenne region, southeastern Belgium (up-left map); the location of the nine local populations are represented in red on an aerial view of the region. On the left of the map, we provide a picture of one marked *Boloria eunomia* butterfly on an inflorescence of *P. bistorta*, its host plant.

*B. eunomia* (formerly known as *Proclossiana eunomia*) is a Holarctic butterfly species occurring in wet meadows and some peat bogs in middle Europe. In this area, the species is a specialist, strictly associated with the bistort *Polygonum bistorta* (Figure 1), its host plant at larval stage and the only source of nectar for adults in the study area. The species is univoltine: it spends the winter as a caterpillar and adults fly around for about one month from late-May to early-July. In Belgium, *B. eunomia* is protected because it suffers, as in other European countries, from the transformation of its habitat (bistort meadows) into improved pastures or spruce *Picea abies* plantations (e.g., [45]). We collected samples in nine local populations in a ~200 km^2^ landscape in the Belgian Ardenne (Figure 1) in 2009, 2010 and 2011 (three successive generations) using both a Capture-Mark-Recapture approach and a genetic sampling approach, described in more details below. The Euclidian distances between the centroid of each site are available in Table S1.

### 2.2. Demographic Modelling: Population Size, Survival and Dispersal from CMR Campaigns

For the three successive years of sampling, each site was visited daily during the flight period (excluding rainy days), and Capture-Mark-Recapture (CMR) sessions performed with a sampling effort normalized according to habitat patch area. We captured butterflies with a net, sexed and marked each of them with a unique identifier using a thin pen on the left hindwing at the first capture (Figure 1). We marked 5481 individuals in total. These CMR data were first used to compute the number of dispersers between each pair of populations over the three years. CMR data were then analysed using Jolly–Seber models, as implemented in the POPAN analysis in MARK software [53]. Based on capture histories of the different individuals recorded in a population, the probability of an individual to be (re)captured, a measure of detectability, is estimated (data per population are given in Table S2), and subsequently used to correct estimates of survival, birth rates, daily and total (seasonal) census population sizes (*N*) [54]. For details of the analytical method, see [45]. To test for the congruence between the ranking of the nine population sizes across the three years (a proxy of global demographic stability), we performed a Kruskal-Wallis rank test between 2009–2010, 2010–2011 and 2009–2011. We finally compiled all recapture data to count the number of dispersers between each pair of populations over the three years. 

### 2.3. Molecular Markers, Genetic Structure and Effective Dispersal

#### 2.3.1. Laboratory Work

Among the 5481 individuals marked during CMR, one leg was preserved from 1217 of them in absolute ethanol and total genomic DNA later extracted using the column version of the DNeasy Blood and Tissue kit (Qiagen, Venlo, The Netherlands). From these 1217 DNA extractions, we multiplexed and amplified by PCR 12 microsatellite loci as described before [55]. Tests for linkage disequilibrium and the presence of null alleles were performed in [55] and showed the presence of a few null alleles that did not significantly impacted population genetic indices. The genotyping was performed on an ABI3730 sequencer (Applied Biosystems, GeT GenoToul platform, Toulouse, France). Fragment sizes for each locus were determined with the GeneMapper software.

#### 2.3.2. Population Structure

Genetic differentiation between all pairs of populations was estimated with Wright’s pairwise fixation index *F_ST_* [56] using Arlequin v.3.5.2.2 [57] for each generation separately. Significance of *F_ST_* values was determined based on 10,000 random permutations, and *p*-values were adjusted with the Benjamini-Yekutieli correction [58]. Genetic clustering of individuals was assessed using the Bayesian clustering method Structure v.2.3.4 [35] including all generations in a full analysis. We used the admixture model, which allows mixed ancestries of individuals, and the correlated allele frequency model (*F* model), which assumes that allele frequencies in different populations are likely to be similar. Five independent runs for each value of *K* (the number of clusters) ranging from one to nine were performed, using 500,000 iterations and a burn-in period of 50,000 steps. To detect the number of clusters that best fit the data, we estimated the rate of change in the log probability of the data between successive *K* values and the corresponding variance of log probabilities [59]. We completed this analysis with a global Analysis of Molecular Variance (AMOVA) performed per generation using Arlequin to determine if the genetic variance was effectively significantly structured by the inter-cluster differences detected with Structure. The *p*-values were calculated using 10,000 permutations. Despite the existence of two significant major genetic clusters, a large part of the genetic variance remained unexplained (see Results). We therefore performed supplementary clustering runs within each first-order cluster to search for significant sub-structuring, and continued the process across hierarchical levels until no more structure was detected [60]. Significant sub-genetic structure was detected with Bayesian assignation, and *F_ST_* methods revealed that genetic structuring was significant at the level of our nine predefined populations (see Results). We thus continued with the genetic analyses for the nine populations separately, thus matching the partition used in the demographic estimates described above.

#### 2.3.3. Isolation by Distance

To test for Isolation By Distance (IBD), that is the effect of Euclidian distance between populations on gene flow, a Mantel test regressing *F_ST_*/(1–*F_ST_*) against the natural logarithm of geographic distance between all pairs of samples is generally performed [61]. However, since measures of *F_ST_* stem from the balance between gene flow on the one hand and genetic drift on the other hand, *F_ST_* estimates cannot be considered a proper proxy for gene flow, especially when population sizes are unequal [30]. We thus corrected *F_ST_* values using the method developed in [31]. For each pair of populations, we first computed the *di* metric of Spatial Heterogeneity in Effective population sizes (*SHNe*) as the sum of the inverse of population census sizes *N* inferred from demographic data. For each generation separately, we then computed the residuals of the linear regression between *F_ST_*/(1–*F_ST_*) and *di* across all pairs of populations, and used a simple Mantel test with 10,000 permutations to assess significance of the relationship between these residuals and the natural logarithm of geographic distances.

#### 2.3.4. Allelic Diversity and Effective Population Sizes

For the three generations and each of the nine populations, the number of alleles (*Na*), the expected (*He*) and observed (*Ho*) heterozygosities, and the *F_IS_* were calculated using Arlequin and averaged over all loci. The number of private alleles (*Np*) was estimated using Convert v.1.31 [62]. The Allelic Richness (*A*) and Private Allelic Richness (*Ap*) based on the minimum sample size were estimated by the rarefaction method implemented in HP-Rare v.1.1. We estimated contemporary effective population sizes (*Ne*) for each generation using the bias-corrected version of the linkage disequilibrium method described in [63] implemented in NeEstimator v.2 [64]. With a sufficient number of microsatellites, sample sizes generally higher than 25 individuals as well as non-overlapping generations, we fulfilled most of the prerequisites of the method. We set the critical threshold value of rare alleles to 0.02 and used the jackknife method to estimate confidence intervals. The significance of correlations between *Ne* (effective population size) and *N* (census population size) estimates for the nine local populations was assessed using a Spearman correlation test for each year separately.

#### 2.3.5. Effective Dispersal and Statistical Comparison with Demographic Dispersal

We determined contemporary dispersal by estimating the number of first-generation dispersers between populations using Geneclass2 [36]. Using Monte Carlo resampling algorithms, the program computes the probability for each individual to be a resident in the population where it was sampled or to descend from a disperser at the preceding generation. We used the Bayesian criterion described in [65] and detection was done using the ratio of the likelihood computed from the population where the individual was sampled over the highest likelihood value among all sampled populations [66]. The probability for an individual to be a resident was computed using the resampling algorithm of [66] with 10,000 simulated individuals and a level of Type I error set to 0.01 as advised by these authors. To assess the relationship between the demographic and the genetic estimates of contemporary dispersal rates, we used a simple Mantel test with 10,000 permutations based on the Spearman’s correlation coefficient between the pairwise numbers of first-generation dispersers inferred from Geneclass2 (genetic matrix) and the pairwise numbers of dispersers inferred from CMR data over the three years (demographic matrix). Significance was assessed by permuting 10,000 times the names of the nine populations in one of the two matrices.

#### 2.3.6. Past Demographic Events

We finally searched for the existence of past demographic events, i.e., bottlenecks and exponential changes in population sizes. We used Bottleneck v.1.2.02 [67] to calculate, for each population and each locus, the distribution of the expected gene diversity from the observed number of alleles given the sample size and assuming mutation-drift equilibrium. In neutral conditions, the number of loci showing *He* excess should be equal to the number of loci showing *He* deficit. Conversely, *He* excess across loci is expected after a bottleneck whereas *He* deficiency is expected after exponential change in population size. Expected *He* under mutation-drift equilibrium was determined by coalescent simulations under the stepwise mutation model (SMM), and the two-phased Mutation model (TPM), with more than one-repeat mutations occurring at frequencies of either 10 and 20%. Wilcoxon sign-rank tests were used to determine significant departures from null distributions, with 10,000 iterations, and they were adjusted with the Benjamini-Yekutieli correction [58]. As a qualitative complement to this analysis, we also used the Bottleneck software to examine the shape of the allele-frequency distribution. L-shaped distributions are expected under mutation-drift equilibrium while mode-shift distributions are expected in cases of bottlenecks [68].

## 3. Results

### 3.1. Demography from CMR Data

**Figure 2:**
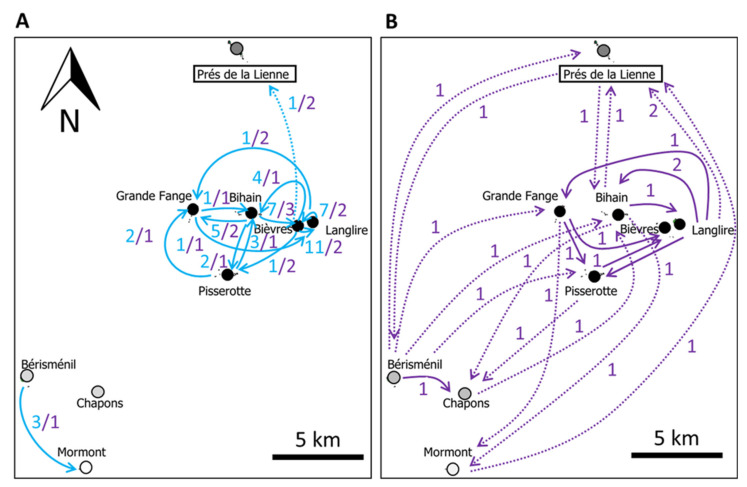
Contemporary dispersal movements from demographic (blue arrows) and genetic (purple arrows) data cumulated over the three sampling years. (**A**) movements directly recorded in the field via Capture-Mark-Recapture (blue), complemented by the number of first-generation effective movements inferred from genetic data (purple) for the same pairs of populations. (**B**) All the other movements inferred from genetic data for other pairs of patches, separated to gain in map clarity. Long-distance movements are symbolized by dashed arrows.

From the 5481 individuals marked during the three years of CMR campaigns, we estimated the total local census population sizes (*N*) of each of the nine populations separately (Table 1). We provide details on male and female population sizes, catchability and survival in Table S2. The Pisserotte and Prés de la Lienne populations were the largest populations; Bérisménil, Bièvres, Bihain and Grand Fange populations had intermediate population sizes; and Chapons, Mormont and Langlire had lower abundances. The ranking of population sizes across years was congruent (no rejection of the null hypothesis, Kruskal-Wallis test χ^2^ = 8, df = 8, *p*-value = 0.433 for each pair of years comparison). Regarding individual dispersal movements, we recorded a total of 49 inter-population movements over the three years, of which only one was considered as a long-distance movement, i.e., more than 5 km (dashed arrow in Figure 2A).

### 3.2. Genetic Structure, Isolation by Distance and Diversity

**Figure 3:**
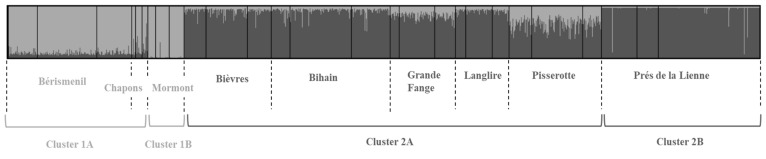
Genetic clustering across the whole metapopulation of *Boloria eunomia* using the three years of sampling. Bayesian clustering shows two major clusters separating Bérismenil, Chapons and Mormont (light grey) from the other populations (dark grey). Each bar represents the membership assignment to the two clusters of each individual. Within each population, vertical black traits separate the three years of sampling in the same order (2009, 2010 and 2011). A second run of analysis in the two clusters reveals the sub-structuring as indicated at the bottom of the figure.

Using the DNA extracted from the legs of 1217 butterflies, the first step of Bayesian clustering revealed the existence of two major genetic clusters repeatedly found across years of sampling (Figure 3, Figure S1A). Pisserotte and Grande Fange were the two populations with the highest mixed ancestry origin, but they were nonetheless well assigned to cluster 2. This was confirmed by the AMOVA analysis, which showed that a significant amount of genetic variance was explained by the among-clusters structure (Table S3). However, this represented only 3.1%, 1.7% and 3.6% of the total genetic variance for 2009, 2010 and 2011 respectively, most of it being explained by suborder structuring. We thus ran again Structure within cluster 1 and 2 separately, and detected sub-structuring distinguishing the Mormont and Prés de la Lienne populations (Figure S1B,C). By continuing the procedure, we detected sub-structuring that was congruent with the delineation of local populations (data not shown). We confirmed this result with *F_ST_* analyses. We detected significant genetic differentiation for each pair of the nine populations sampled in the metapopulation in at least one out of the three years (Table S4). We detected a significant IBD pattern for the three years, with correlation coefficients between genetic distances and Euclidian distances of 0.502 (*p* < 0.0001), 0.536 (*p* < 0.0001) and 0.467 (*p* = 0.049) for 2009, 2010 and 2011 respectively after *SHNe* correction (Figure S2). Usual genetic diversity indices averaged across the 12 loci are presented in Table 2. Populations had rather similar levels of genetic diversity, except the Mormont and Chapons populations, which were less diversified. Overall, populations had a recurrent deficit in heterozygotes across years as indicated by significant positive *F_IS_* values.

### 3.3. Comparison between Estimates of Population Sizes and Dispersal

We estimated contemporary effective population sizes (Table 1) using NeEstimator and found that they were significantly correlated with estimates of census population sizes for the three years (Spearman’s *rho* = 0.28 in 2009, *p* = 0.050; 0.83 in 2010, *p* = 0.008; and 0.83 in 2011, *p* = 0.015). Over all years and populations, the *N*e/*N* ratio was 0.38. At this contemporary timescale, we also detected significant correlation between the number of effective dispersal movements at the preceding generation (we detected 46 movements with GenClass2) and the number of movements observed by CMR, when pooling the three years (Spearman’s *rho* = 0.547, *p* < 0.0001, Figure 2A,B). Despite this significant correlation, it is noteworthy that genetic data revealed a number of long-distance dispersal movements that were not captured by CMR data (Figure 2B). 

### 3.4. Past Demographic Events

In a last analysis, we searched for genetic footprints of past demographic events. By comparing the number of microsatellite loci presenting deficit or excess in heterozygotes to the expected number under mutation-drift equilibrium, we were unable to detect any demographic event after correction for multiple testing (Table S5). The Chapons population was excluded from the analysis because of a too low number of individuals to obtain reliable outputs. This result was confirmed by the qualitative exploration of the shape of the allele-frequency distribution, which was normal for every year in every population, except for Grande Fange in 2009 (Table S5). This sample presented a shifted distribution toward too many loci with heterozygote deficiency, revealing a potential population expansion.

## 4. Discussion

Understanding the dynamics of natural metapopulations at relevant spatial and temporal scales is of prime importance to accurately feed both theoretical eco-evolutionary models and conservation plans [69]. Adding new demographic and genetic data to a well-studied European metapopulation of *B. eunomia*, we were able to highlight the general stability of the genetic structure and effective sizes of its local populations. Building on a trans-generational sampling at both demographic and genetic levels, we highlighted congruence in the estimates of dispersal rates and local population sizes across years, suggesting that these two approaches can fruitfully be used to infer metapopulation functioning. This also suggests that, at least in some cases, ecological parameters can be used as reliable proxies of evolutionary parameters, and the other way round. Based on the comparison between demographic and genetic parameters, we discuss below the ecological modalities that underlie this metapopulation equilibrium (two first sections). We also emphasize crucial methodological limits to our study (third section), which could affect the congruence between demographic and genetic parameters, notably due to the lack of integration of the effect of landscape structure on dispersal.

### 4.1. Metapopulation Functioning: An Integrative Story in B. eunomia over Space and Time

We estimated several important metrics summarizing the structure and dynamics of a natural butterfly metapopulation aiming at evaluating its stability. First, the ranking of local population sizes as estimated from intensive CMR campaigns (i.e., census adult population sizes) was consistent across generations. This means that, despite large observed fluctuations in population size between years in many of the local populations [47,48], they all fluctuated with some synchrony overtime. Genetic estimates of population sizes (i.e., effective population sizes) confirmed this finding as they were correlated with census adult population sizes. Such congruent ranking of population sizes in a metapopulation network can be interpreted as a global control of demographic fluctuations, with putatively low temporal variation in the mean *Npop_i_*/*Npop_j_* ratio. Comparing census and effective populations sizes, we found the mean *Ne/N* ratio to be 0.38 over years and populations. This is higher than the value usually observed in many systems (~0.1–0.2, [70]). We suggest that this could be due to features of the life history of *B. eunomia* in this region allowing a large proportion of the individuals to effectively reproduce, as opposed to cases where reproductive success is very high for a few individuals, or very low for the majority. In *B. eunomia*, males mate multiple times along their life (females only once), increasing their chance to reproduce at least once. Besides, the large availability of the host plant, combined with continuous egg laying in small batches over the whole female lifetime, increases reproductive success by creating a situation where mortality risks for eggs and larvae are spread over space and time. Although relatively high given the census population sizes, *Ne* were systematically far below the threshold of 1000 reproductive individuals ensuring high evolutionary potential to local populations [70]. Accordingly, we detected a recurrent heterozygosity deficit at the local population scale, most probably resulting from inbreeding depression. Such indicators would suggest the metapopulation to be at risk of future extinction. Searching for past demographic events from genetic data, we were yet unable to detect any drastic reduction in local population sizes in neither of the three generations, which could suggest a progressive erosion rather than an abrupt collapse in population sizes. Such scenario could explain our limited ability to detect recent changes in population sizes through molecular methods. Putting all these elements together, we suggest that the studied metapopulation remained stable over the last decades despite small local population sizes, with a low probability of undetected recent population size changes. Accordingly, stochastic local population extinctions have very rarely been observed in populations monitored for decades now, even when their local population sizes were recurrently small like Mormont. 

Second, we observed a stable genetic structure of the metapopulation over the three years of sampling. Bayesian clustering (Structure analysis) revealed a first level of genetic partition separating the southern populations of Bérismenil, Mormont and Chapons from the six northern other populations. The Pisserotte and Grande Fange populations had the highest mixed ancestry among all populations, which agrees with their central positions in the network. However, Grande Fange had higher level of private allelic richness and *F_ST_* values than Pisserotte. While these two populations probably exchange a lot with the other populations of the network, Grande Fange could be more isolated than Pisserotte, and could have particular local dynamics allowing private genetic diversity to be maintained. Grande Fange could have experienced a recent founding effect followed by an expansion (as suggested by its deficit in heterozygous loci), but could also have acted as a past local refugia [71] or function nowadays as a sink [72], the two hypotheses being non-exclusive. Such genetic particularities could reflect the existence of (dis)assortative mating or local selection. On the contrary, Pisserotte had the highest census and effective population sizes over years, which suggests it is a hub for immigrants coming almost equally from the two clusters. A second Structure run revealed significant sub-structuring, with on the one hand the distinction of Mormont, the southernmost population among the three composing cluster 1, and on the other hand the distinction of the Prés de la Lienne, the northernmost population among the six composing cluster 2. By continuing the process, we detected genetic partition at the level of the nine local populations, which was confirmed by the general significance of *F_ST_* values. The IBD analysis confirmed the suspected impact of geographic distance on genetic differentiation: about 25% of the genetic variance between populations could be attributed to Euclidian distances. It is noteworthy that both genetic structure and IBD were highly consistent over the three generations, meaning that the above-described temporal stability in the ranking of population sizes goes along with equilibrium in genetic structure. Nevertheless, the time lag between demographic processes and the genetic response could have hindered our ability to detect recent changes in population structure, even with fast evolving markers such as microsatellites [34]. Future sampling could help bring this possible scenario to light.

Third, we measured contemporary (inter-annual pool of effective first-generation dispersers versus inter-annual pool of direct movements) estimates of dispersal rates. There was a significant correlation between the demographic and genetic approaches, showing that dispersal movements were more frequent between close populations. This pattern agrees with the significant IBD pattern. As expected, CMR campaigns detected less long-distance dispersal movements (more than 5 km) than the genetic approach [12,73]. We yet detected similar numbers of dispersal movements (49 through CMR versus 46 through microsatellite analysis), which suggests that the individual monitoring of *B. eunomia* dispersal movements overestimated the real number of effective short-distance movements (Figure 2), as expected from [12]. Overall, dispersal estimates showed that populations are all connected by dispersal movements but with frequencies contingent upon their geographic distance. However, the significance of *F_ST_* values indicates that effective dispersal is not high enough to genetically homogenize the metapopulation. 

Metapopulation functioning might rely on source/sink dynamics, the permanent dispersal of individuals from a source population of good quality to a receiving population with demographic deficit living in habitats of poorer quality [74]. On the long-term, source/sink dynamics may lead to the demographic stabilization of the overall system and could explain some aspects of the described metapopulation equilibrium. However, the qualitative analysis of dispersal movements did not show any obvious source/sink pattern. This suggests that strong dispersal asymmetry might not be predominant in the functioning of this *B. eunomia* metapopulation, although we cannot exclude a role of weak source/sink patterns, like the putative case of Grande Fange (a population harboring a high level of private alleles, see above). We hypothesize that the observed stability probably results from other eco-evolutionary processes and discuss this possibility hereafter. 

### 4.2. Eco-Evolutionary Perspectives: Controlled Ecological Fluctuations Lead to Long-Term Equilibrium at the Metapopulation Scale

The focal metapopulation has been surveyed for decades, and we accumulated knowledge about various eco-evolutionary processes that might further explain its stability. Population parameters are often subject to oscillations as a result of the confrontation of intrinsic (phenotypes) to extrinsic (environments) factors (see review in [75]), and *B. eunomia* is no exception. For instance, its larvae are attacked by a tiny hymenopteran parasitoid, which abundance regulates the butterfly local population size [45,76,77]. Besides, local population sizes are correlated with climatic factors (temperature and humidity) acting differently over the year according to the life history stages of the butterfly [46]. Together with the host- and food-plant abundance and other factors (see [40,78] for detailed reviews), the habitat quality varies over time on a yearly basis and across space on a few hundred meters [46]. The habitat of the butterfly may thus be seen as a moving mosaic of patches of low and high quality that support different adult densities [48]. Adult males and females move differently between high and low quality patches. Due to male harassment, females of *B. eunomia* emigrate seeking for patches with low male density, while males emigrate seeking for patches with high female density [41]. Besides, male harassment in combination with life-history characteristics (differential reproductive success, detectability by predators) probably explains the long-term maintenance of female color polymorphism, i.e., existence of an andromorph wing coloration in some females [79]. Such selective patterns may control local demographic fluctuations and trait variability and suggest a long-adaptive history of *B. eunomia* to its local environment, including its predictable oscillations. Habitat quality and butterfly density within local populations are fine-grained at the scale of a few hundred meters [46], which should favor local efficient response to microhabitat fluctuations as experienced by the species in this area over thousands of generations. Adult movements among patches of different quality prevent in turn strong spatial synchrony. Altogether these two processes (fine grained adaptation and adult movements within and among local populations) mitigate the risk of metapopulation collapse caused by synchronous environmental variations. Thus, we argue that, in concert with the control of ecological fluctuations at the local scale, the current rate of dispersal and gene flow (strongly dependent upon geographic distance between populations) is a key mechanism conferring stability to the system. Indeed, when dispersal is sufficiently high to prevent very high consanguinity and local extinctions, and sufficiently low to avoid region-wide synchrony and genetic pool homogenization, such equilibrium metapopulations should maintain on the long-term [80]. However, the persistence of such metapopulations at equilibrium in cases of catastrophic events is not guaranteed if the amplitude of the catastrophe exceeds the regulatory eco-evolutionary feedbacks between local adaptation and dispersal. This hypothesis could unfortunately be formally tested now in our studied metapopulation. The large Prés de la Lienne local population has indeed recently gone extinct, very likely as a direct consequence of poorly prepared reintroduction of beavers (*Castor spp.*) in Belgium. The construction of beaver dams along the Lienne river created frequent and long-lasting floods during the winter in the wet meadows inhabited by *Boloria eunomia*. Although this is still an unproven but likely hypothesis, these floods should lead to very high mortality rates of the diapausing caterpillars, susceptible to cause this large local population to vanish over a couple of years: from over 1000 individuals in 2010 and 2011, only 6 males were captured in 2016, and none in the next years. We hope to be able to estimate the impact of the Prés de la Lienne extinction on the stability of the whole metapopulation in the forthcoming years.

### 4.3. Methodological Perspectives: Congruence between Demographic and Genetic Estimates of Dispersal

The ability of demographic approaches such as CMR to estimate effective dispersal is still a matter of debate. It supposedly suffers from several biases: difficulties in the acquisition of field data, adequation of demographic models to natural situations, definition of dispersal movements, scale effects, etc. [12,32,33,81]. However, we here found a noticeable congruence between demographic and genetic approaches (*r* = 0.5). We highlight below a few critical points that could improve the match between demographic and genetic approaches in *B. eunomia* (and beyond) in future works.

Despite intensive field work covering the whole flight period in a well-studied butterfly system, we were unable to capture the vast majority of long-distance dispersal movements using CMR data. This recurrent difficulty in the monitoring of individual movements has strong consequences on the ability to construct appropriate dispersal kernels, where long-distance movements play a key role, but also to define the exact contours of natural metapopulations. In *B. eunomia*, unsampled distant populations nonetheless connected by rare dispersal movements to our metapopulation could serve as reservoirs feeding the general stability of the whole-system, i.e., undetected source/sink dynamics. The eco-evolutionary mechanisms we proposed above to explain equilibrium (fine-grained local adaptation and dispersal) could in this case be less predominant. Underestimation of long-distance dispersal and overestimation of short-distance movements with the demographic approach raise one important question about the origin of the uncommon variance between the two approaches (~75% in our case). Can we attribute most of this unexplained variance to this CMR campaign effect, or should we look for other explanations? A number of other methodological biases can be mentioned. They include for instance the ability of genetic methods to effectively detect first-generation dispersers, incomplete genetic sampling, or asymmetric reproductive success. It will be difficult to tackle these issues in our current dataset. An easy improvement would nonetheless be the use of more numerous molecular markers, such as SNPs, to get more accurate genetic estimates.

Finally, we observed a strong IBD pattern, which makes sense given that long-distance dispersal movements are less numerous than short-distance movements in the studied metapopulation. Nonetheless, ~75% of the genetic variance remains unexplained by the Euclidian distance between populations. *Boloria eunomia* presents distinct behaviors when encountering different matrix types [82]. Although beyond the scope of this study, we will probably need to incorporate functional connectivity indices in a landscape genetic approach to better explain our observed pattern of genetic differentiation.

## 5. Conclusions

There is no doubt that classical metapopulations do exist, but they might not be as widespread as generally supposed, because extinction/recolonization cycles are not necessary characteristics of metapopulation functioning [6,7]. This study on *B. eunomia* provides an example of a metapopulation where destabilizing agents like inter-generational fluctuations in population sizes seem to be controlled by a long adaptive history of the species to its dynamic local environment, including the evolution of appropriate rates of dispersal. In such a case, population extinction should be the result of rare catastrophic events, whose consequences on short-term dynamics and long-term stability are of prime interest to study in this butterfly of conservation concern. In our study system, genetic and demographic approaches provided congruent estimates of dispersal rates. This comfortable situation may not hide the necessity to integrate, e.g., functional connectivity to fully capture the functioning of our metapopulation. Indeed, a non-negligible part of the variance in genetic differentiation remained unexplained. We hope that other case studies of such stable ‘unclassical natural metapopulations’ will be available to test for the generality of the mechanisms we have proposed to explain metapopulation equilibrium in *B. eunomia*.

## Figures and Tables

**Table 1:** Estimates of population sizes for each year of sampling. *N* = census population size recorded from Capture-Mark-Recapture data, *Ne* = effective population size inferred from microsatellite data analyzed with NeEstimator with their confidence intervals (*CI Ne*).

	2009			2010			2011		
Population	*N*	*Ne*	*CI Ne*	*N*	*Ne*	*CI Ne*	*N*	*Ne*	*CI Ne*
Bérismenil	196	58.6	27.4–410.1	191	35	23.6–54.5	235	102	39.8–∞
Chapons	14	NA	NA	13	1.9	1.1–6.1	8	14.6	1.7–∞
Mormont	25	4.7	1.7–32.2	30	9.9	3.9–24.6	56	12.8	4.4–59.1
Bièvres	195	50.5	19.2–∞	259	44.8	26.4–92.5	304	46	26.9–109.9
Bihain	136	22.4	12.3–54.3	419	64.4	40.6–118.5	289	111	52.4–990.9
Grande Fange	70	17	3–∞	166	55.8	26.8–240.2	336	113	34–∞
Langlire	57	91.2	12.5–∞	101	56.5	23–∞	99	95.3	23.8–∞
Pisserotte	978	34.3	14.2–522.8	1428	142.6	72.7–657.9	1139	NA	84.9–∞
Prés de la Lienne	291	46.4	26.8–105.1	940	57.7	24.6–∞	1131	122	75.4–241.1

**Table 2:** Genetic diversity. *n* = genetic sample size, *Na* = number of alleles, *Np* = number of private alleles, *A* = Allelic richness, *Ap* = Private allelic richness, *Ho* = observed heterozygosity, *He* = expected heterozygosity, significant (< 0.05) *p*-values for *F_IS_* values are in bold in the last column.

Population	Year	*n*	*Na*	*Np*	*A*	*Ap*	*Ho*	*He*	*F_IS_*	*p*-Value
*Bersimenil*	2009	47	5.3	2	3.54	0.18	0.44	0.64	0.17	**0.0003**
	2010	97	5.1	0	3.69	0.16	0.47	0.61	0.08	**0.002**
	2011	56	5.2	0	3.64	0.11	0.52	0.67	0.09	**0.004**
*Chapons*	2009	6	2.9	0	2.5	0.09	0.6	0.61	−0.15	0.6
	2010	11	3	0	2.69	0.05	0.41	0.45	−0.01	0.59
	2011	9	3.5	1	3.13	0.14	0.43	0.61	0.24	**0.01**
*Mormont*	2009	13	3	2	2.74	0.27	0.46	0.57	0.008	0.51
	2010	22	3.6	0	2.94	0.18	0.34	0.58	0.26	**<0.0001**
	2011	24	3.6	0	2.84	0.17	0.39	0.55	0.16	**0.006**
*Bièvres*	2009	36	5.6	1	3.74	0.12	0.43	0.68	0.18	**<0.0001**
	2010	67	6.4	2	3.91	0.15	0.48	0.68	0.15	**<0.0001**
	2011	38	6.1	2	3.8	0.15	0.48	0.69	0.13	**0.0003**
*Bihain*	2009	31	5.6	2	3.79	0.21	0.46	0.69	0.18	**<0.0001**
	2010	99	6.7	2	3.87	0.18	0.47	0.68	0.13	**<0.0001**
	2011	63	6.4	3	3.81	0.18	0.48	0.7	0.15	**<0.0001**
*Grande Fange*	2009	15	4.4	4	3.52	0.4	0.49	0.64	0.13	0.47
	2010	57	6.5	5	4.06	0.21	0.48	0.69	0.17	**<0.0001**
	2011	34	5.8	4	3.76	0.21	0.47	0.68	0.12	**0.002**
*Langlire*	2009	17	5.1	2	3.69	0.17	0.45	0.68	0.19	**0.004**
	2010	42	5.4	2	3.99	0.09	0.46	0.68	0.19	**<0.0001**
	2011	28	5.6	0	3.75	0.09	0.41	0.68	0.18	**<0.0001**
*Pisserotte*	2009	36	5.3	2	3.56	0.06	0.47	0.67	0.09	**0.03**
	2010	83	6	0	3.94	0.15	0.49	0.69	0.13	**<0.0001**
	2011	31	5.3	0	3.74	0.04	0.49	0.69	0.15	**0.0008**
*Prés Lienne*	2009	57	5.1	2	3.31	0.12	0.42	0.6	0.14	**<0.0001**
	2010	35	4.6	1	3.27	0.11	0.41	0.62	0.15	**0.0003**
	2011	163	5.4	3	3.3	0.08	0.41	0.6	0.23	**<0.0001**

## Data Availability

Data is contained within the article and Supplementary Materials.

## Supplementary Materials

The following are available online at https://www.mdpi.com/2073-4425/12/3/362/s1, Figure S1: Determination of the number of clusters in the Structure analysis, Figure S2: Isolation by distance patterns per year, Table S1: Euclidean distance between pairs of population in meters, Table S2: Demographic results from CMR modelling, Table S3: AMOVA analyses considering clusters 1 and 2 as the unit of genetic partitioning, Table S4: Fst values and significance, Table S5: Results of the detection of past demographic events using Bottleneck. Raw microsatellite data are provided as a word file entitled ‘*microsat_data_structure_format*’.
