# Supplementary material for: Congruent Genetic and Demographic Dispersal Rates in a Natural Metapopulation at Equilibrium
Source: Genes (Basel). 2021 Mar 3;12(3):362. doi: 10.3390/genes12030362 (PMC7999359; doi:10.3390/genes12030362)
Supplement: Supplementary file 1 [file genes-12-00362-s001.pdf]

Supplementary Material

Congruent genetic and demographic dispersal rates in a natural metapopulation at equilibrium

Delphine Legrand, Michel Baguette, Jérôme Prunier, Quentin Dubois, Camille Turlure, Nicolas Schtickzelle

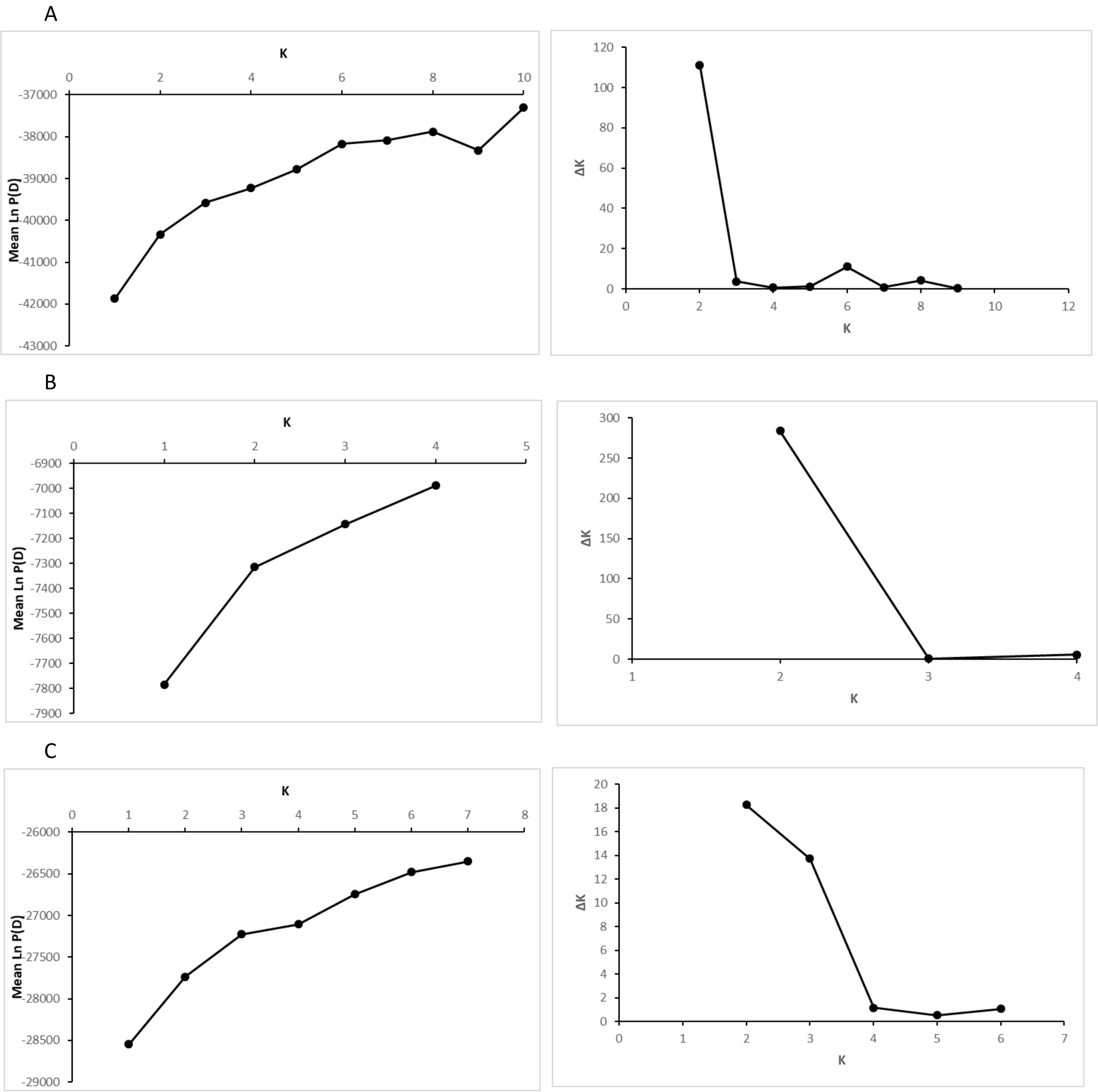

<u>Figure S1:</u> Determination of the number of clusters in the Structure analysis. In each left plot, the y-axis displays the mean log probability of the data [Ln Pr(*X*I*K*)] over five runs as a function of *K* in the x-axis. In each right plot, the y-axis displays *ΔK* values as a function of *K*, calculated according to the method of Evanno et al. (2005). A = first step of the determination of the number of clusters (test for *K* ranging from 1 to 9), *i.e.*, at the level of the whole dataset. B **=** Determination of the number of clusters within the Bérismenil/Chapons/Mormont cluster (cluster 1, test for *K* ranging from 1 to 4). C = Determination of the number of clusters within the Bièvres/Bihain/Grande Fange/Langlire/Pisserotte/Prés de la Lienne cluster (cluster 2, test for *K* ranging from 1 to 7).

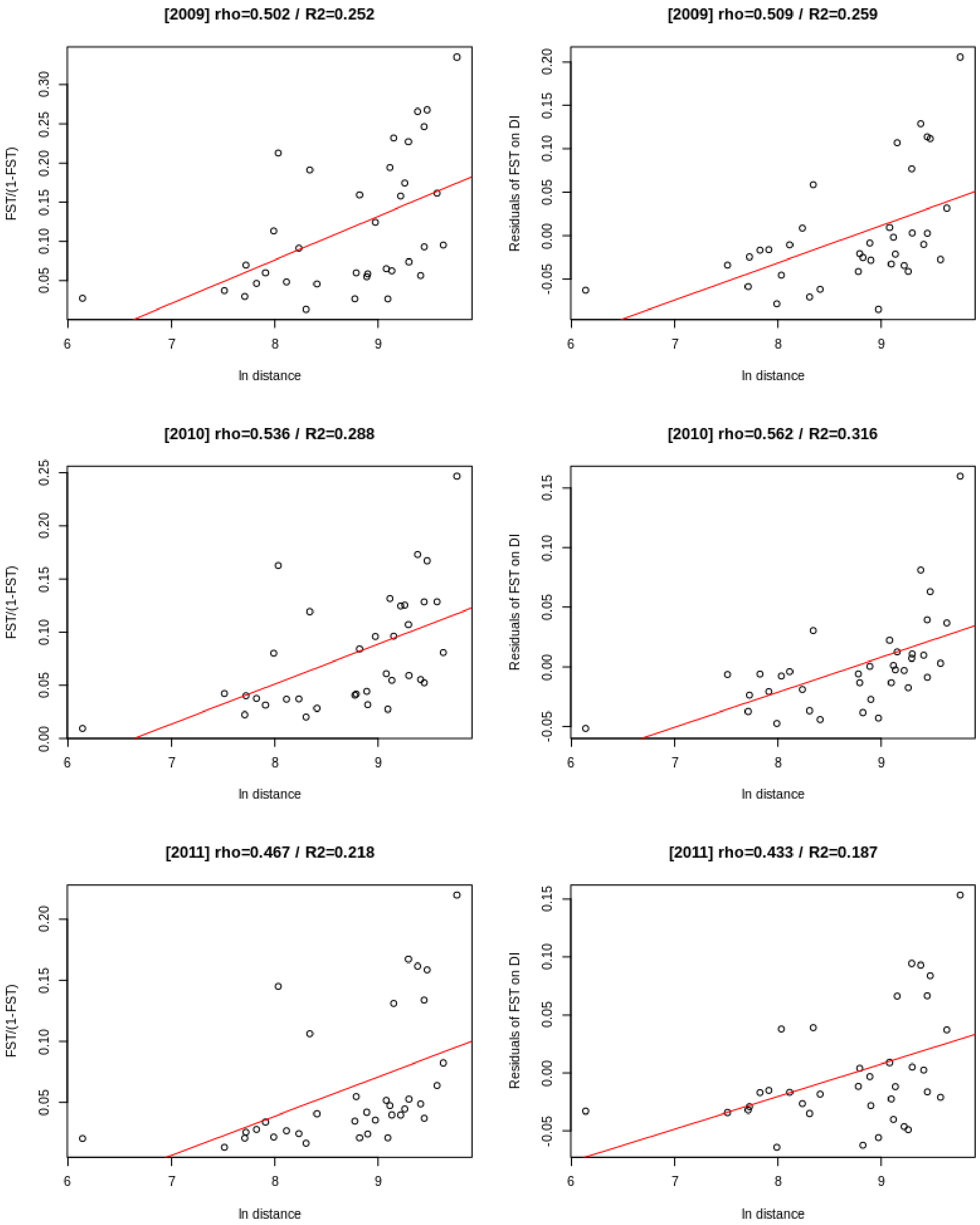

<u>Figure S2:</u> Isolation by distance patterns per year. On the left graph is represented the relationship between the log of the natural logarithm of the Euclidian distance and genetic differentiation (*Fst*/(1-*Fst*)) without SHNe correction, while the same relationship is illustrated on the right panel applying the SHNe correction. Each graph represents one year of sampling (2009 to 2011 from the top to the bottom), and the linear regression line is represented in red.

	Bérismenil	Chapons	Mormont	Bièvres	Bihain	Grande Fange	Langlire	Pisserotte	Prés de la Lienne
Bérismenil	0								
Chapons	2868	0							
Mormont	4064	3085	0						
Bièvres	12221	10100	12659	0					
Bihain	10846	9027	11825	1957	0				
Grande Fange	9232	7824	10817	4075	2140	0			
Langlire	12492	10313	12812	537	2397	4495	0		
Pisserotte	8921	6792	9455	3321	2488	270	3579	0	
Prés de la Lienne	15314	14356	17395	7276	6472	6572	7547	8779	0

Table S1: Euclidean distance between pairs of population in meters.

year	population	sex	marked	population size	apparent daily survival	catchability
2009	Bérismenil	F	78	138	0.88	0.31
2009	Bérismenil	M	37	58	0.91	0.32
2009	Bièvres	F	48	88	0.89	0.36
2009	Bièvres	M	63	107	0.91	0.30
2009	Bihain[1]	F	17	50	0.90	0.27
2009	Bihain[1]	M	36	86	0.89	0.27
2009	Chapons[2]	F	4	8	0.94	.
2009	Chapons[2]	M	5	6	0.96	.
2009	Grande fange	F	14	23	0.91	0.31
2009	Grande fange	M	21	47	0.83	0.27
2009	Langlire[2]	F	9	16	0.88	.
2009	Langlire	M	27	41	0.93	0.26
2009	Prés de la Lienne	F	68	117	0.98	0.13
2009	Prés de la Lienne	M	130	174	0.87	0.47
2009	Mormont	F	12	14	0.93	0.65
2009	Mormont	M	10	11	0.93	0.52
2009	Pisserotte	F	172	462	0.86	0.19
2009	Pisserotte	M	357	516	0.92	0.31
2010	Bérismenil	F	67	95	0.93	0.45
2010	Bérismenil	M	84	96	0.86	0.71
2010	Bièvres	F	69	145	0.87	0.31
2010	Bièvres	M	88	114	0.88	0.54
2010	Bihain	F	89	175	0.84	0.35
2010	Bihain	M	120	244	0.87	0.27
2010	Chapons[3]	F	5	5	0.95	0.85
2010	Chapons[3]	M	6	8	0.98	0.26
2010	Grande fange	F	28	51	0.79	0.32
2010	Grande fange	M	74	115	0.88	0.37
2010	Langlire[4]	F	22	48	0.86	.
2010	Langlire[4]	M	24	53	0.86	.
2010	Prés de la Lienne	F	213	472	0.90	0.19
2010	Prés de la Lienne	M	322	468	0.88	0.36
2010	Mormont	F	12	15	0.95	0.41
2010	Mormont	M	10	15	0.92	0.43
2010	Pisserotte	F	332	677	0.93	0.19
2010	Pisserotte	M	546	751	0.92	0.34
2011	Bérismenil	F	73	120	0.88	0.38
2011	Bérismenil	M	85	115	0.83	0.57
2011	Bièvres	F	87	167	0.91	0.23
2011	Bièvres	M	108	137	0.87	0.55
2011	Bihain	F	75	143	0.90	0.23
2011	Bihain	M	96	146	0.82	0.46
2011	Chapons[3]	F	2	2	1.00	0.51
2011	Chapons[3]	M	6	6	0.98	0.71
2011	Grande fange	F	95	194	0.79	0.30
2011	Grande fange	M	93	142	0.81	0.45
2011	Langlire[5]	F	15	37	0.71	0.32
2011	Langlire[5]	M	25	62	0.71	0.32

2011	Prés de la Lienne	F	308	498	0.93	0.22
2011	Prés de la Lienne	M	439	633	0.90	0.34
2011	Mormont[6]	F	14	21	0.89	0.42
2011	Mormont[6]	M	21	35	0.84	0.42
2011	Pisserotte	F	318	583	0.88	0.20
2011	Pisserotte	M	402	556	0.84	0.40

<u>Table S2:</u> Demographic results from CMR modelling

[1]Catchability forced to be equal between sexes.

[2]Too few data so population size unreliable, fixing catchability at 0.30.

[3]Too few data so population size unreliable

[4]No female recaptured, so fixing female survival equal to male, and catchability at 0.3

[5]No male never missed on capture (*i.e.*, all recaptures of each male are done on consecutive samplings), then fixing male catchability equale to female

A

2009	% variance	*F-indices*	p-value
Among clusters	3.06	0.031	**0.039**
Among populations within groups	6.18	0.064	**<10-5**
Within populations	90.76	0.092	**<10-5**

B

2010	% variance	*F-indices*	p-value
Among clusters	1.74	0.017	**0.011**
Among populations within groups	4.3	0.044	**<10-5**
Within populations	93.97	0.06	**<10-5**

C

2011	% variance	*F-indices*	p-value
Among clusters	3.6	0.036	**0.011**
Among populations within groups	3.95	0.04	**<10-5**
Within populations	92.45	0.075	**<10-5**

Table S3: AMOVA analyses considering clusters 1 and 2 as the unit of genetic partitioning. A=2009, B=2010 and C=2011.

A

2009	Bérismenil	Chapons	Mormont	Bièvres	Bihain	Grand Fange	Langlire	Pisserotte	Prés de la Lienne
Bérismenil	-	**<10-5**	**<10-5**	**<10-5**	**<10-5**	**<10-5**	**<10-5**	**0.0012**	**<10-5**
Chapons	0.10183	-	**0.0003**	**<10-5**	**<10-5**	**0.0003**	**<10-5**	**<10-5**	**<10-5**
Mormont	0.16056	0.17527	-	**<10-5**	**<10-5**	**<10-5**	**<10-5**	**<10-5**	**<10-5**
Bièvres	0.05351	0.13625	0.19770	-	**0.0002**	0.15	0.023	**<10-5**	**<10-5**
Bihain	0.06891	0.16274	0.21002	0.03585	-	0.021	**<10-5**	**<10-5**	**0.001**
Grand Fange	0.05890	0.11070	0.18504	0.01313	0.02878	-	**0.007**	**0.0001**	**0.0003**
Langlire	0.08504	0.14854	0.21123	0.02660	0.06537	0.04373	-	**<10-5**	**<10-5**
Pisserotte	0.02582	0.13733	0.18820	0.04609	0.04434	0.05665	0.08360	-	**<10-5**
Prés de la Lienne	0.08696	0.13895	0.25098	0.05211	0.02596	0.05662	0.05546	0.06130	-

B

2010	Berismenil	Chapons	Mormont	Bièvres	Bihain	Grand Fange	Langlire	Pisserotte	Prés de la Lienne
Bérismenil		**<10-5**	**<10-5**	**<10-5**	**<10-5**	**<10-5**	**<10-5**	**<10-5**	**<10-5**
Chapons	0.07406		**<10-5**	**<10-5**	**<10-5**	**<10-5**	**<10-5**	**<10-5**	**<10-5**
Mormont	0.10651	0.14000		**<10-5**	**<10-5**	**<10-5**	**<10-5**	**<10-5**	**<10-5**
Bièvres	0.05250	0.11067	0.11373		**<10-5**	**0.003**	0.039	**<10-5**	**<10-5**
Bihain	0.05593	0.11618	0.14737	0.04047		**<10-5**	**<10-5**	**<10-5**	**<10-5**
Grand Fange	0.05182	0.08748	0.09677	0.01985	0.02188		**<10-5**	**<10-5**	**<10-5**
Langlire	0.04975	0.11129	0.14319	0.00936	0.03851	0.02746		**<10-5**	**<10-5**
Pisserotte	0.02670	0.07752	0.08773	0.03549	0.03617	0.03028	0.03573		**<10-5**
Prés de la Lienne	0.07462	0.11380	0.19791	0.04234	0.03904	0.04003	0.03063	0.05748	

C

2011	Berismenil	Chapons	Mormont	Bièvres	Bihain	Grand Fange	Langlire	Pisserotte	Prés de la Lienne
Bérismenil		0.078	**<10-5**	**<10-5**	**<10-5**	**<10-5**	**<10-5**	**0.0011**	**<10-5**
Chapons	0.02125		**<10-5**	0.153	**0.005**	0.023	0.016	0.134	**0.0007**
Mormont	0.09606	0.12663		**<10-5**	**<10-5**	**<10-5**	**<10-5**	**<10-5**	**<10-5**
Bièvres	0.04644	0.03820	0.11792		**0.009**	0.01	**0.0075**	**0.0013**	**<10-5**
Bihain	0.05001	0.04518	0.13913	0.01308		**0.0006**	**<10-5**	**<10-5**	**<10-5**
Grand Fange	0.03827	0.03446	0.14321	0.01634	0.02040		**<10-5**	**0.001**	**<10-5**
Langlire	0.03587	0.04263	0.13690	0.02012	0.02507	0.03915		**0.0039**	**0.0007**
Pisserotte	0.02064	0.02063	0.11577	0.02616	0.02722	0.03295	0.02394		**<10-5**
Prés de la Lienne	0.07609	0.06005	0.18011	0.04030	0.03367	0.05191	0.02365	0.04909	

Table S4: ***Fst* values and significance**. The bottom left right diagonal gives *Fst* values, the upper right diagonal gives *p*-values, with significant values after Benjamini-Yekutieli correction in bold. A=2009, B=2010 and C=2011.

mutational model	*year*	*Bérismenil*	*Mormont*	*Bièvres*	*Bihain*	*Grande Fange*	*Langlire*	*Pisserotte*	*Prés de la Lienne*
SMM	2009	0.57	0.24	0.91	0.79	0.622	0.518	0.85	0.266
	2010	0.176	0.301	0.034	0.042	0.301	0.569	0.733	0.898
	2011	0.176	0.966	0.339	0.151	0.733	0.791	0.91	0.129
TPM80%	2009	0.092	0.012	0.23	0.204	0.092	0.677	0.013	0.85
	2010	0.0425	0.077	0.151	0.339	0.233	0.011	0.042	0.638
	2011	0.042	0.24	0.423	0.424	0.203	0.11	0.077	0.622
TPM90%	2009	0.301	0.0537	0.424	0.339	0.204	1	0.38	0.85
	2010	0.052	0.11	0.677	0.733	0.569	0.077	0.176	0.831
	2011	0.042	0.413	0.91	0.91	0.47	0.203	0.233	1
Mode-Shift	2009	normal	normal	normal	normal	**shifted**	normal	normal	normal
	2010	normal	normal	normal	normal	normal	normal	normal	normal
	2011	normal	normal	normal	normal	normal	normal	normal	normal

Table S5: Results of the detection of past demographic events using Bottleneck. For each population and each year, the *p*-value for the detection of heterozygosity excess or deficit is presented. None of these *p*-values were significant after correction for multiple testing (*p*-value after Benjamini-Yekutieli correction = 0,01011). Mode-Shift gives an indication of the shape of the allele-frequency distribution.
